# Differential diagnosis of precocious puberty in girls during the COVID-19 pandemic: a pilot study

**DOI:** 10.1186/s12887-023-04009-x

**Published:** 2023-04-20

**Authors:** Huihui Sun, Yi Qian, Naijun Wan, Lili Liu

**Affiliations:** 1 Pediatrics Department, Beijing Jishuitan Hospital, Beijing, China; 2 Acupuncture Department, Beijing Jishuitan Hospital, Beijing, China

**Keywords:** Precocious puberty, Idiopathic premature thelarche (IPT), Central precocious puberty (CPP), LH, Bone age (BA), Predicted adult height (PAH), Overweight, Obesity

## Abstract

**Background:**

To investigate the differential diagnosis of girls aged 6 to 8 years with idiopathic premature thelarche (IPT) and central precocious puberty (CPP) during the COVID-19 pandemic. We explored predicted adult height (PAH) discrepancy to guide appropriate diagnosis and treatment.

**Methods:**

From January 2020 to December 2021, Chinese girls aged 6 to 8 years with precocious puberty were recruited. They were divided into IPT and CPP groups. Clinical characteristics, including height, weight, body mass index (BMI), basal luteinizing hormone (LH), oestradiol, uterine length and volume, follicle numbers (d > 4 mm) and bone age (BA) were recorded. We analysed differential diagnosis and PAH discrepancy in both groups. Binary logistic regression analysis was used to explore risk factors for CPP, and receiver operating characteristic (ROC) curves were generated to evaluate the diagnostic value of related indexes.

**Results:**

Sixty patients, including 40 girls with IPT and 20 girls with CPP, were recruited. The prevalence of overweight and obesity in the entire cohort was 25% (15/60) and was significantly higher in IPT than CPP, 32.5% (13/40) vs. 10% (2/20), respectively (*P*=0.045). There were significant differences in LH, uterine volume, follicle numbers and BA (*P*<0.05). The impaired PAH of IPT and CPP was 0.01 ± 1.19 SD and 0.62 ± 0.94 SD with significant differences (*P*=0.047). Logistic regression analysis showed that LH and follicle numbers were independent risk factors for CPP. The ROC curve showed that the area under the curve (AUC) of LH and follicle numbers were 0.823 and 0.697. The sensitivity and specificity of LH with a cut off of 0.285 IU/L were 78.9% and 77.8%. The sensitivity and specificity of follicle numbers with a cut off of 3.5 were 89.5% and 52.8%.

**Conclusion:**

The prevalence of overweight and obesity in 6- to 8-year-old girls with precocious puberty was high. Auxological data should not be used in the differential diagnosis of IPT and CPP. Basal LH above 0.285 IU/L and follicle numbers greater than 4 were important features suggestive of CPP. PAH was impaired in individuals with CPP, but it was not impaired in individuals with IPT.

## Introduction

Precocious puberty is a common endocrinological disease. According to its pathogenesis, precocious puberty is classified into peripheral precocious puberty (PPP), central precocious puberty (CPP) and benign variants, including isolated premature thelarche (IPT) [1, 2]. CPP in girls is commonly defined as the development of secondary sexual characteristics caused by premature activation of the hypothalamic-pituitary-gonadal (HPG) axis before 8 years of age [3–5]. IPT is defined as the appearance of isolated breast development before 8 years in girls without other signs of puberty [6, 7]. IPT is a benign disorder; however, in some patients, IPT can be induced by HPG axis activation, leading to CPP [6, 8]. CPP causes psychological disturbances and physical damage, such as short adult stature, early age at menarche, and risks of cardiovascular diseases [3, 9]. From the perspective of cartilage growth plate procedural ageing, early detection of CPP and prompt treatment are more effective in improving final adult height (FAH) [10]. Therefore, it is particularly crucial to differentiate IPT from CPP for prognosis and management.

Pubertal development varies notably at the individual level. A trend towards earlier pubertal onset in girls has been observed [11]. The initiation of puberty depends on genetic alterations and neurosecretory activity and is influenced by nutrition, exercise, and emotional and environmental factors [11, 12]. CPP is caused by early activation of the HPG axis with both FSH and LH pulsatile secretion, which cause ovarian oestrogen secretion and breast development [13]. The pathophysiology of IPT is complex due to partial HPG axis initiation mainly by follicle-stimulating hormone (FSH) secretion, lack of luteinizing hormone (LH) secretion, breast tissue sensitivity, or excessive oestrogens release from multiple origins [14]. LH is considered a powerful marker of gonadal axis activation; however, there was a lack of agreement on basal LH cut-offs for CPP diagnosis in different centres [3]. The gonadotropin-releasing hormone (GnRH) stimulation is the gold standard for CPP diagnosis [15]. In girls with breast development, excessive stimulation tests could be a waste of medical resources and result in psychological burdens on the patients [1]. Therefore, it is important to explore basal LH cut-offs to distinguish between IPT and CPP in our centre [3, 15].

During the novel coronavirus COVID-19 pandemic, the prevalence of precocious puberty in girls has increased across all social classes [14, 16, 17]. Few studies have been performed on the clinical features of girls with precocious puberty during this unique period [1, 16]. This study focused on the clinical characteristics of Chinese girls aged 6 to 8 years with breast development during the COVID-19 pandemic [17]. We investigated growth and development data, LH, oestradiol (E2), pelvic ultrasound, bone age (BA) and predicted adult height (PAH) damage in girls with precocious puberty, providing a basis of differential diagnosis in IPT and CPP.

## Materials and methods

### Participants

This prospective observational study was performed in the paediatric department of Beijing Jishuitan Hospital during the COVID-19 pandemic from January 2020 to December 2021. The project was approved by the Ethics Committee of Beijing Jishuitan Hospital. Clinical data from 60 Chinese girls aged 6 to 8 years with precocious puberty were collected. Data from their physical records included chronological age (CA) at visit, onset of breast budding, height, weight, body mass index (BMI), breast stage, LH, E2, uterine length, uterine volume, number of follicles with diameter greater than 4 mm (d > 4 mm), and BA. Uterine volume = length*width*thickness*0.5233. PAH was calculated according to BA assessed by a radiologist and a paediatrician using the Greulich and Pyle (G-P) atlas and growth curves of Chinese girls [18]. Mid-parental height (MPH) = (paternal height + maternal height-13)/2. PAH discrepancy = MPH - PAH.

The diagnostic criteria for CPP in girls were as follows [19]: (1) Breast development before 8 years; (2) Linear growth acceleration; (3) Advanced bone age by more than one year; (4) Uterine and ovary enlargement and multiple follicles with diameters greater than 4 mm; and (5) Hypothalamic-pituitary-gonadal axis activation. The exclusion criteria were as follows (6): (1) exogenous oestrogen intake; (2) gonadal tumour, adrenal disease or other organic diseases; and (3) chromosomal abnormalities and genetic diseases.

### GnRH stimulation test

The GnRH stimulation test was performed using subcutaneous administration of the GnRH analogue triptorelin (Ferring AG, Saint-Prex, Switzerland). The dosage was 2.5 µg/kg with a maximum dose of 100 µg. Then, blood samples were drawn at the 0′, 30′, 60′, and 90′ time points to examine LH and follicle-stimulating hormone (FSH) concentrations. Serum LH and FSH were measured by immunochemiluminescent assay (ICMA) using a Beckman UniCel DxI800 automatic chemiluminescence analyser. The GnRH stimulation test was performed in patients with breast development and LH > 0.1–0.2 IU/L. CPP was diagnosed when the peak value of LH was greater than 5.0 U/L, and the LH/FSH ratio was greater than 0.6.

### Statistical analysis

Statistical analyses were performed using the Statistical Packages for the Social Sciences (SPSS) version 24.0 software (Chicago, USA). To compare the differences between IPT and CPP, independent-samples t tests were used to analyze the significant differences in normally distributed measurement data, which was expressed as M ± SDS. The Mann‒Whitney U test was used for nonnormally distributed data, which are expressed as [M (QR)]. Binary logistic regression using the forward likelihood ratio (LR) method was applied for CPP risk factor analysis. Receiver operating characteristic (ROC) curves were constructed to evaluate the sensitivity and specificity of the risk factors. Youden’s J index (sensitivity + specificity-1) was used to determine the optimal diagnostic cut-off points based on the ROC curves. The prevalence of overweight and obesity in both groups was analysed using a chi-square (χ^2^) test. Data from these patients were further categorized and analysed according to BMI. The cut-off for statistical significance was accepted as *P* < 0.05.

## Results

The cohort included 40 individuals with IPT (66.7%, 40/60) and 20 individuals with CPP (33.3%, 20/60). Clinical features are shown in Table 1. In the IPT group, 17 patients completed GnRHa stimulation tests, and 12 patients had isolated breast development with LH < 0.1–0.2 IU/L. Eleven patients completed follow-up of more than 3 to 6 months, and symptoms of breast budding were found to have receded. In the CPP group, 13 patients completed GnRHa stimulation tests, and 4 patients were diagnosed with CPP based on elevated LH and oestradiol (E2), uterine enlargement, and multiple follicles. The other 3 cases were followed up for more than 3 to 6 months, and their secondary sexual characteristics developed progressively. The final diagnosis was based on clinical manifestations, GnRHa stimulation tests and follow-up.

**Table 1:** Clinical features of 6- to 8-year-old girls with IPT and CPP

Items	IPT(n = 40)	CPP(n = 20)	Statistcs	P values
CA (years)	7.50 (6.42,7.79)	7.38(7.27,7.81)	Z=-0.998	0.318
MPH (cm)	160.55 ± 3.98	160.38 ± 3.84	t = 0.163	0.871
Birth weight (kg)	3242.63 ± 404.36	3268.42 ± 500.89	t=-0.212	0.833
Age at maternal menarche (years)	12.68 ± 1.11	12.76 ± 1.16	t=-0.281	0.780
Age at onset (years)	7.00 (6.12,7.42)	7.33 (6.92,7.67)	Z=-2.098	0.036*
Height (cm)	127.06 ± 6.90	128.35 ± 3.08	t=-1.000	0.321
Height Z score	0.68 ± 1.05	0.58 ± 0.67	t = 0.730	0.469
Height Z score – MPH Z score	0.68 ± 0.86	0.53 ± 0.88	t = 0.610	0.544
Weight (kg)	27.18 ± 5.85	26.41 ± 2.94	t = 0.554	0.582
Weight Z score	0.95 ± 1.29	0.58 ± 0.67	t = 1.448	0.153
BMI	16.72 ± 2.63	16.01 ± 1.56	t = 1.116	0.269
BMI Z score	0.81 ± 1.30	0.46 ± 0.79	t = 1.124	0.266
Overweight/obesity (%)	32.5	10	χ2 = 4.030	0.045*
LH	0.11 (0.00,0.27)	0.50 (0.24,0.82)	Z=-3.716	0.000*
E2 (pmol/L)	12.09 (0.00,47.67)	54.51 (0.00,115.13)	Z=-1.782	0.075
Uterine length	3.43 ± 0.57	3.61 ± 0.44	t=-1.172	0.247
Uterine volume	1.20 (0.84,1.84)	1.74 (1.34, 2.69)	Z=-2.358	0.018*
Follicle number (d > 4 mm)	3.00 (1.00,5.00)	5.00 (4.00,6.00)	Z=-2.407	0.016*
BA	7.84 ± 1.41	8.65 ± 0.76	t=-2.893	0.005*
BA-CA(years)	0.69 ± 1.01	1.20 ± 0.76	t=-2.006	0.050
PAH Z score	-0.01 ± 1.00	-0.65 ± 0.66	t = 2.582	0.012*
PAH Z score – MPH Z score	0.01 ± 1.19	0.62 ± 0.94	t=-2.032	0.047*

* *P* < 0.05

CA, chronological age; MPH, mid-parental height; BMI, body mass index; BA, bone age; PAH, predicted adult height

The prevalence of overweight and obesity in the entire cohort was 25% (15/60) and was significantly higher in the IPT group than in the CPP group: 32.5% (13/40) vs. 10% (2/20), respectively (χ^2^ = 4.030, *P* = 0.045). There were no significant differences in CA at visit, MPH, birth weight, age at maternal menarche, height, weight, BMI, uterine volume or BA advancement (BA-CA) between the groups (*P*≥0.05). There were significant differences in age at onset, LH, uterine volume, number of follicles (d > 4 mm), BA, PAH and PAH discrepancy between the groups (*P* < 0.05).

**Fig. 1:**
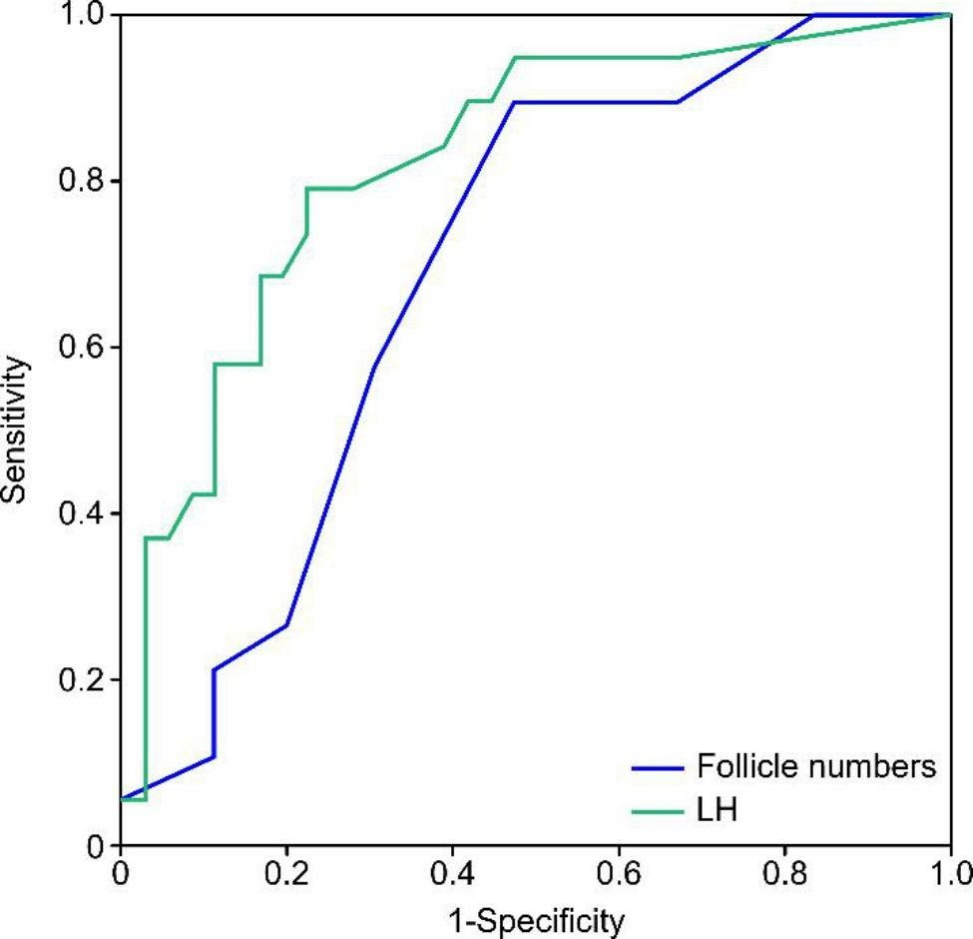
The diagnostic predictive values of basal LH and follicle number for CPP

Binary logistic regression analysis showed that basal LH levels (*OR* = 13.958, CI: 1.917-101.621) and numbers of follicles (d > 4 mm) (*OR* = 1.486, CI: 1.081–2.041) were two independent risk factors for CPP. The area under the curve (AUC) of LH levels was 0.823 (95% CI: 0.706–0.940, *P* = 0.000). The AUC for follicle number was 0.697 (95% CI: 0.557–0.836, *P* = 0.017). The maximum Youden’s J index was found for a cut-off point of 0.285 IU/L at the LH level and follicle number of 3.5. The sensitivity and specificity of basal LH with a cut off of 0.285 IU/L were 78.9% and 77.8%, respectively. The sensitivity and specificity of follicle numbers with a cut off of 3.5 were 89.5% and 52.8%, respectively. These results are shown in Fig. 1.

There were 27 IPT cases and 18 CPP cases with normal BMI. There were no significant differences in CA, MPH, birth weight, age at maternal menarche, age at onset, height, weight, BMI, uterine length, BA advancement, or PAH discrepancy between the two groups (*P*≥0.05). There were significant differences in LH, uterine volume, follicle numbers, BA, and PAH between the groups (*P* < 0.05). These results are shown in Table 2.

**Table 2:** Clinical features of girls with IPT and CPP with normal BMI and overweight/obesity

Items	IPT with normal BMI (n = 27)	CPP with normal BMI (n = 18)	IPT with Overweight/Obesity(n = 13)	Statistcs 1	*P*1 value	Statistcs 2	*P*2 value
CA (years)	7.50(6.58,7.83)	7.37(7.31,7.77)	6.58(6.34,7.59)	Z=-0.209	0.834	Z=-0.956	0.339
MPH (cm)	160.74 ± 3.97	160.61 ± 3.83	160.15 ± 4.11	t = 0.109	0.914	t=-0.433	0.668
Birth weight (kg)	3139.07 ± 379.36	3241.18 ± 519.39	3457.69 ± 381.25	t=-0.753	0.456	t=-2.484	0.018*
Age at maternal menarche (years)	12.63 ± 1.17	12.74 ± 1.23	12.77 ± 1.03	t=-0.287	0.775	t=-0.368	0.715
Age at onset (years)	7.00(6.25,7.42)	7.33(7.05,7.71)	6.33(6.09,7.25)	Z=-1.521	0.128	Z=-1.143	0.253
Height (cm)	126.97 ± 7.28	128.42 ± 3.21	127.25 ± 6.31	t=-0.914	0.367	t=-0.122	0.904
Height Z score	0.49 ± 1.02	0.58 ± 0.54	1.06 ± 1.06	t=-0.386	0.701	t=-1.621	0.113
Height Z score – MPH Z score	0.46 ± 0.74	0.56 ± 0.90	1.14 ± 0.96	t=-0.416	0.680	t=-2.465	0.018*
Weight (kg)	25.06 ± 5.85	25.80 ± 2.39	26.50 ± 2.99	t=-0.677	0.502	t=-3.842	0.000*
Weight Z score	0.95 ± 1.29	0.49 ± 0.61	0.59 ± 0.68	t=-0.664	0.510	t=-5.829	0.000*
BMI	15.44 ± 1.26	15.62 ± 1.00	19.39 ± 2.76	t=-0.512	0.611	t=-4.927	0.000*
BMI Z score	0.15 ± 0.80	0.29 ± 0.62	2.18 ± 1.03	t=-0.612	0.544	t=-6.872	0.000*
LH	0.10 (0.00,0.28)	0.42 (0.21,0.66)	0.14(0.01,0.27)	Z=-3.217	0.001*	Z=-0.706	0.480
E2 (pmol/L)	13.98(0.00,46.28)	54.5(0.00,102.77)	7.13(0.00,60.82)	Z=-1.645	0.100	Z=-0.062	0.950
Uterine length	3.36 ± 0.64	3.64 ± 0.46	3.61 ± 0.28	t=-1.500	0.142	t=-1.597	0.120
Uterine volume	1.22 (0.81,1.73)	1.74 (1.35,2.82)	1.02(0.82, 1.85)	Z=-2.128	0.033*	Z=-0.749	0.454
Follicle number (d > 4 mm)	3.00 (1.00,6.00)	5.00 (4.00,6.50)	3.50(0.75,5.00)	Z=-1.928	0.048*	Z=-0.339	0.735
BA	7.82 ± 1.53	8.53 ± 0.71	7.89 ± 1.19	t=-2.119	0.041*	t=-0.145	0.886
BA-CA(years)	0.57 ± 1.07	1.09 ± 0.77	0.92 ± 0.86	t=-1.789	0.081	t=-1.026	0.311
PAH Z score	0.01 ± 1.02	-0.52 ± 0.55	-0.05 ± 1.01	t=-2.286	0.027*	t = 0.184	0.855
PAH Z score – MPH Z score	0.02 ± 1.20	0.55 ± 0.96	-0.03 ± 1.22	t=-1.556	0.127	t = 0.125	0.901

* *P* < 0.05

Statistics 1 and *P*1 indicate the difference between IPT and CPP with normal BMI. Statistics 2 and *P*2 indicate the difference between IPT with normal BMI and overweight/obesity

Among girls with IPT, 27 had a normal BMI and 13 had overweight or obesity. There were no significant differences in CA, MPH, age at maternal menarche, age at onset, height, LH, E2, uterine length and volume, follicle numbers (d > 4 mm), BA, BA advancement, PAH or PAH discrepancy (*P* ≥ 0.05). There were significant differences in birth weight (*P* = 0.018) and height Z score minus MPH Z score (*P* = 0.018). These results are shown in Table 2.

## Discussion

We investigated the differential diagnosis of 6- to 8-year-old girls with IPT and CPP and their influence on PAH in the unique period [13]. There were no significant differences in MPH, birth weight or age at maternal menarche between the two groups, which implied similar genetic backgrounds. There were no significant differences in height, weight, or BMI. Therefore, anthropometric parameters should not be used to distinguish CPP from IPT [7]. Our study demonstrated that LH, uterine volume, follicle numbers and BA exhibited important differences between IPT and CPP. Binary regression analysis showed that basic LH levels and follicle numbers were two independent risk factors for CPP. Patients with LH exceeding 0.285 IU/L and more than 4 follicles were more likely to have CPP. Our study proposes that girls with LH above 0.285 IU/L should further undergo the GnRH stimulation test to confirm CPP diagnosis, greatly reducing stimulation numbers and alleviating medical burdens. These results were consistent with those of previous studies, in which basal LH levels above 0.255 IU/L or 0.3 IU/L (ICMA) were considered indicative of puberty [1, 5, 6]. Girls with breast development and LH levels below 0.285 IU/L should be followed up and monitored to rule out CPP.

Recent studies have shown that the increased prevalence of precocious puberty in girls was associated with a high incidence of overweight and obesity [6]. In this study, the prevalence of overweight and obesity in individuals with precocious puberty was high [1, 20]. During the COVID-19 pandemic, increased energy intake, reduced physical activity, excessive sedentary behaviour, and exposure to various stressors have contributed to an increase in childhood overweight and obesity [20, 21]. Childhood adiposity increases risks of cardiovascular disease and endocrine disturbances, especially early pubertal development in girls [22]. Most of the girls with breast development were diagnosed with IPT. It was demonstrated that IPT was more common than CPP in girls aged 6 to 8 years with breast development and overweight and obesity. Our results partially indicated that earlier breast development was associated with higher BMI [12]. The potential mechanisms of correlation between nutritional excess and pubertal advance in girls may include leptin-mediated stimulation, aromatase overactivation or IGF-1 increasement [11, 23].

Different from previous studies, BMI Z scores and height Z scores in individuals with IPT were both higher than those in individuals with CPP, although with no significant differences [14]. The height Z scores were synchronized with BA advancement, so PAH was not affected in IPT patients. Because of accelerated skeletal maturation, PAH in CPP patients was significantly decreased [24]. Nevertheless, in girls with normal BMI and precocious puberty, the height Z scores of CPP patients were higher than those of IPT patients due to accelerated linear growth [2]. However, PAH in the CPP group was also significantly lower than that in the IPT group. Therefore, IPT, with or without overweight/obesity, had no obvious impact on PAH, but girls with CPP had impaired PAH [2]. GnRHa treatment should be tailored to prevent pubertal progression and bone age acceleration to improve final adult height (FAH) in CPP patients [25].

Compared with girls with IPT and a normal BMI, the height Z score minus the MPH Z score was significantly higher in girls with IPT and overweight/obesity, in whom BA advancement was dominant. These results also showed that overweight and obesity can cause growth acceleration and BA advancement [20, 26]. There was no difference in PAH between individuals with normal BMI and those with overweight/obesity. The PAH in girls with IPT and overweight or obesity was not higher than their target height. In addition, their birth weights were significantly higher than those of girls with a normal BMI. It was suggested that fetal weight could affect childhood BMI and that high birth weight was significantly associated with childhood obesity [20, 27, 28]. Management of the fetal weight gain was considered to be beneficial to prevent childhood obesity and early puberty [27].

As a prospective observational study, we need to further expand the sample size to confirm the findings of these study. In addition, PAH based on BA has bias, and follow-up of FAH is necessary [2].

## Conclusions

In summary, the prevalence of overweight and obesity in girls with precocious puberty during the COVID-19 pandemic was high. Auxological data should not be used in the differential diagnosis between IPT and CPP in 6- to 8-year-old girls. Basal serum LH levels and follicle numbers are important indexes for the differential diagnosis of CPP. LH above 0.285 IU/L and more than 4 follicles were important features suggestive of CPP. PAH was impaired in CPP patients, but it was not impaired in IPT patients.

## Data Availability

The original contributions presented in the study are included in the article. Further inquiries can be directed to the corresponding author.

## Abbreviations

PPP: Peripheral precocious puberty
CPP: Central precocious puberty
IPT: Isolated premature thelarche
HPG: Hypothalamic-pituitary-gonadal
FAH: Final adult height
FSH: Follicle-stimulating hormone
LH: Luteinizing hormone
GnRH: Gonadotropin-releasing hormone
E2: Oestradiol
BA: Bone age
PAH: Predicted adult height
CA: Chronological age
BMI: Body mass index
MPH: Mid-parental height
ICMA: Immunochemiluminescent assay
SPSS: Statistical Packages for the Social Sciences
LR: Likelihood ratio
ROC: Receiver operating characteristic
AUC: Area under the curve
FAH: Final adult height
